# Type II BMP and activin receptors BMPR2 and ACVR2A share a conserved mode of growth factor recognition

**DOI:** 10.1016/j.jbc.2022.102076

**Published:** 2022-05-26

**Authors:** Kit-Yee Chu, Anjali Malik, Vijayalakshmi Thamilselvan, Erik Martinez-Hackert

**Affiliations:** Department of Biochemistry and Molecular Biology, Michigan State University, East Lansing, Michigan, USA

**Keywords:** TGF-β, bone morphogenetic protein, BMP, BMPR2, activin, ACVR2A, signal transduction, pulmonary arterial hypertension, crystal structure, BMP, bone morphogenetic protein, BRE, BMP responsive element, CHO, Chinese hamster ovary, CM, conditioned medium, EMEM, Eagle’s minimum essential medium, GBD, GF-binding domain, GDF, growth and differentiation factor, GF, growth factor, HEK293, human embryonic kidney cells, NCS, noncrsytallographic symmetry, PAH, pulmonary arterial hypertension, RU, responsive units, SEC, size-exclusion chromatography, SBE, SMAD binding element

## Abstract

BMPR2 is a type II Transforming Growth Factor (TGF)-β family receptor that is fundamentally associated with pulmonary arterial hypertension (PAH) in humans. BMPR2 shares functional similarities with the type II activin receptors ACVR2A and ACVR2B, as it interacts with an overlapping group of TGF-β family growth factors (GFs). However, how BMPR2 recognizes GFs remains poorly understood. Here, we solved crystal structures of BMPR2 in complex with the GF activin B and of ACVR2A in complex with the related GF activin A. We show that both BMPR2 and ACVR2A bind GFs with nearly identical geometry using a conserved hydrophobic hot spot, while differences in contacting residues are predominantly found in loop areas. Upon further exploration of the GF-binding spectrum of the two receptors, we found that although many GFs bind both receptors, the high-affinity BMPR2 GFs comprise BMP15, BMP10, and Nodal, whereas those of ACVR2A are activin A, activin B, and GDF11. Lastly, we evaluated GF-binding domain BMPR2 variants found in human PAH patients. We demonstrate that mutations within the GF-binding interface resulted in loss of GF binding, while mutations in loop areas allowed BMPR2 to retain the ability to bind cognate GFs with high affinity. In conclusion, the *in vitro* activities of BMPR2 variants and the crystal structures reported here indicate biochemically relevant complexes that explain how some GF-binding domain variants can lead to PAH.

Cells communicate to regulate development, maintenance, and regeneration of tissues throughout the lifespan of all multicellular organisms (1). Transforming Growth Factor-β (TGF-β) signaling pathways provide a means for such cell-to-cell communication and tissue fate specification in animals (2, 3, 4). Their role in tissue formation, maintenance, and repair is fundamentally linked with clinically relevant pathologies, including cardiovascular diseases, musculoskeletal disorders, and cancers, making TGF-β pathways key targets for clinical intervention (5, 6).

In mammals, TGF-β pathways comprise over 35 secreted growth factors (GFs), seven “type I” and five “type II” serine/threonine transmembrane kinase receptors, five R-SMAD transcription factors, and many additional accessory factors, including coreceptors, antagonists, and cotranscription factors (7). At the most basic level, TGF-β pathways are activated when a dimeric GF forms a signaling complex with both receptor types, triggering a phosphorylation cascade from receptors to R-SMADs that results in R-SMAD–mediated transcriptional responses (2, 3). Among type II receptors, TGFβR2 and AMHR2 only interact functionally with a select number of GFs, whereas ACVR2A, ACVR2B, and BMPR2 are promiscuous and can form signaling and nonsignaling complexes with an overlapping group of over 30 GFs, including homodimeric and heterodimeric activins, inhibins, bone morphogenetic proteins (BMPs), growth and differentiation factors (GDFs), Nodal, and Lefty (8, 9, 10, 11).

Structural and biochemical studies have revealed that the GF-binding domain (GBD) of both type I and type II receptors adopt a similar, “three-finger toxin fold”, where the “fingers” refer to three pairs of antiparallel β-strands that form an extended β-sheet (12, 13). However, in spite of their significant structural homology, receptors interact with GFs in distinct ways (14). Among type II receptors, TGFβR2 binds GFs *via* an outer β-strand (15, 16). By contrast, ACVR2A and ACVR2B contact GFs *via* a conserved hydrophobic hot spot localized at the center of the β-sheet (17, 18, 19, 20, 21). AMHR2 also binds its cognate GF *via* a centrally located hydrophobic hot spot but uses a distinct set of aliphatic amino acids that confers AMHR2 its unique specificity (22). Less is known about BMPR2. Structures of unbound BMPR2 have been solved (23). Combined with mutagenesis data (24, 25), they indicate that BMPR2 interacts with GFs like ACVR2A/B. Yet despite this knowledge, many questions about the molecular basis of BMPR2-GF recognition, specificity determination, and interacting GFs remain unanswered (23, 26, 27, 28, 29, 30).

Notably, mutations in the *BMPR2* gene are profoundly associated with pulmonary arterial hypertension (PAH) (31), a highly morbid disorder characterized by vascular remodeling with progressive obliteration of the lung microvascular system (32, 33). Loss-of-function *BMPR2* variants are the most common genetic factor in hereditary forms of PAH, implicating deficient BMPR2 signaling in its pathogenesis (34). Nonetheless, missense mutations in the BMPR2-GBD have also been identified (31). What role these variants play in PAH pathogenesis is unclear. To determine how BMPR2 and its GBD variants interact with GFs, we solved the crystal structure of BMPR2-GBD in complex with activin B, a GF that can bind BMPR2 (28, 35) and that signals *via* BMPR2 in some cell types (29, 36). To identify molecular differences in GF recognition, we solved the structure of ACVR2A-GBD in complex with activin A and compared how the two receptors interact with these homologous GFs. To establish GF–receptor affinity and activity preferences, we tested binding of different GFs to soluble BMPR2-Fc and ACVR2A-Fc fusion proteins and evaluated the inhibitory potency of these fusion proteins against their targets. Finally, to define the consequences of BMPR2-GBD mutations identified in PAH patients, we evaluated how variants impact the receptor–GF interaction.

## Results

### BMPR2 and ACVR2A employ an analogous GF-binding mode

**Figure 1:**
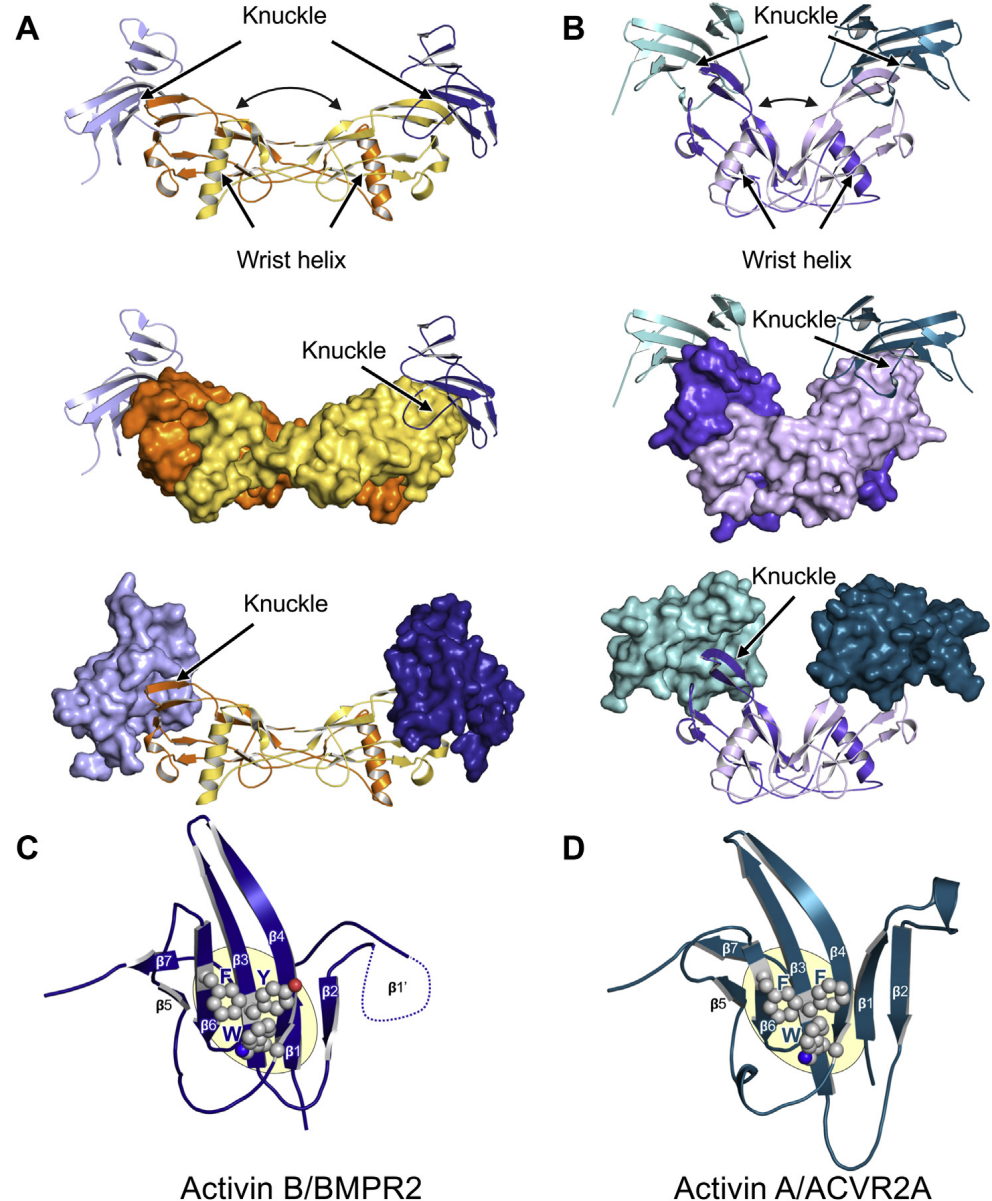
**Crystal structures of the BMPR2–activin B and ACVR2A–activin A complexes.***A*, BMPR2–activin B complex. Activin B protomers are colored *gold* and *orange*, and BMPR2 GBD protomers are shown in *dark* and *light blue*. *B*, ACVR2A–activin A complex. Activin A protomers are colored *light* and *dark purple*, and ACVR2A GBD protomers are shown in *light* and *dark teal*. The *top panels* show all moieties as *ribbon* diagrams, and the *middle panels* show surface representations of the GF moiety. The *bottom panels* show surface representations of the receptors. *Arrows* point out the knuckle epitope where receptors interact with GFs. *C*, BMPR2 and *D*, ACVR2A as seen in complex with their respective GFs. β-strands are labeled to emphasize the “three-finger toxin fold”. The “fingers” are formed by β1–β2 (finger 1), β3–β4 (finger 2), and β5–β6 (finger 3). Conserved hydrophobic patch residues are shown as *spheres*. BMP, bone morphogenetic protein; GBD, GF-binding domain; GF, growth factor.

Figure 1 shows the crystal structures of BMPR2-GBD–activin B and ACVR2A-GBD–activin A complexes. We obtained crystals of the complexes by combining purified GF with approximately 1.2 M excess purified receptor GBD. All proteins were expressed individually in Chinese hamster ovary (CHO) cells, purified to homogeneity, and deglycosylated as needed. We collected diffraction data to a resolution of 3.45 Å and 3.14 Å, respectively, and solved both structures by molecular replacement and multiple rounds of manual rebuilding. For the ACVR2A complex, we used previous crystal structures as search models (37). We solved the BMPR2 complex by placing the activin B protomer structure predicted by AlphaFold (38) first. We then positioned a BMPR2 model lacking loop regions manually and improved its placement with rigid body refinement (23). Notably, previous structures and AlphaFold models were essential for building and refinement of the BMPR2 complex as the resolution of these crystals was limited (For Data collection and refinement statistics, see Table S1, for examples of electron density see Figs. S1 and S2). The asymmetric unit of the BMPR2 complex comprised three receptor and three GF protomers, where all biological dimers were related by crystallographic symmetry. The asymmetric unit of the ACVR2A complex contained four receptor and four GF protomers, where all biological dimers were related by noncrystallographic symmetry (NCS).

BMPR2 binds activin B at its “knuckle epitope” (Fig. 1*A*) (39), a convex surface on the GF that is formed by a set of antiparallel β-strands or “fingers” (40). This binding mode is similar to that between ACVR2A/B and other GFs, as can be seen both in the ACVR2A-activin A structure shown here (Fig. 1*B*) and other ACVR2A/B complexes (17, 18, 19, 20, 21). One significant difference between the two complexes is the relative orientation of GF protomers within a GF dimer (Fig. 1, *A* and *B*). These differences can be attributed to flexibility of the activin dimer interface, which leads activin A to adopt a closed conformation and activin B an extended conformation.

BMPR2 adopts the three-finger toxin fold seen in other TGF-β family receptors with minor variations in β-strand length, flexibility, and orientation (Fig. 1, *C* and *D*). Its GF-binding interface is similar to that of ACVR2A and consists of a concave surface that harbors a hydrophobic hot spot containing three conserved aromatic residues, Y67, W85, and F115, at its center (Fig. 1, *C* and *D*). The buried surface areas of the BMPR2–activin B and ACVR2A–activin A complexes are 710 Å^2^ and 658 Å^2^, respectively (Table 1).

**Table 1:** Protein–protein interaction surface analysis

Protein 1	Protein 2	Interaction	Copy #	BSA (Å^2^)	σBSA (Å^2^)
Activin B–BMPR2 complex					
INHBB	INHBB	GF dimer	2	1518	24
INHBB	BMPR2	GF-receptor	3	710	65
INHBB	INHBB	Lattice	2	1041	138
Activin A–ACVR2A complex					
INHBA	INHBA	GF dimer	2	1346	9
INHBA	ACVR2A	GF-receptor	4	658	17

Abbreviations: BSA, buried surface area; σBSA, SD BSA.

### GF dimer shape is determined by ‘wrist helix’ positioning

**Figure 2:**
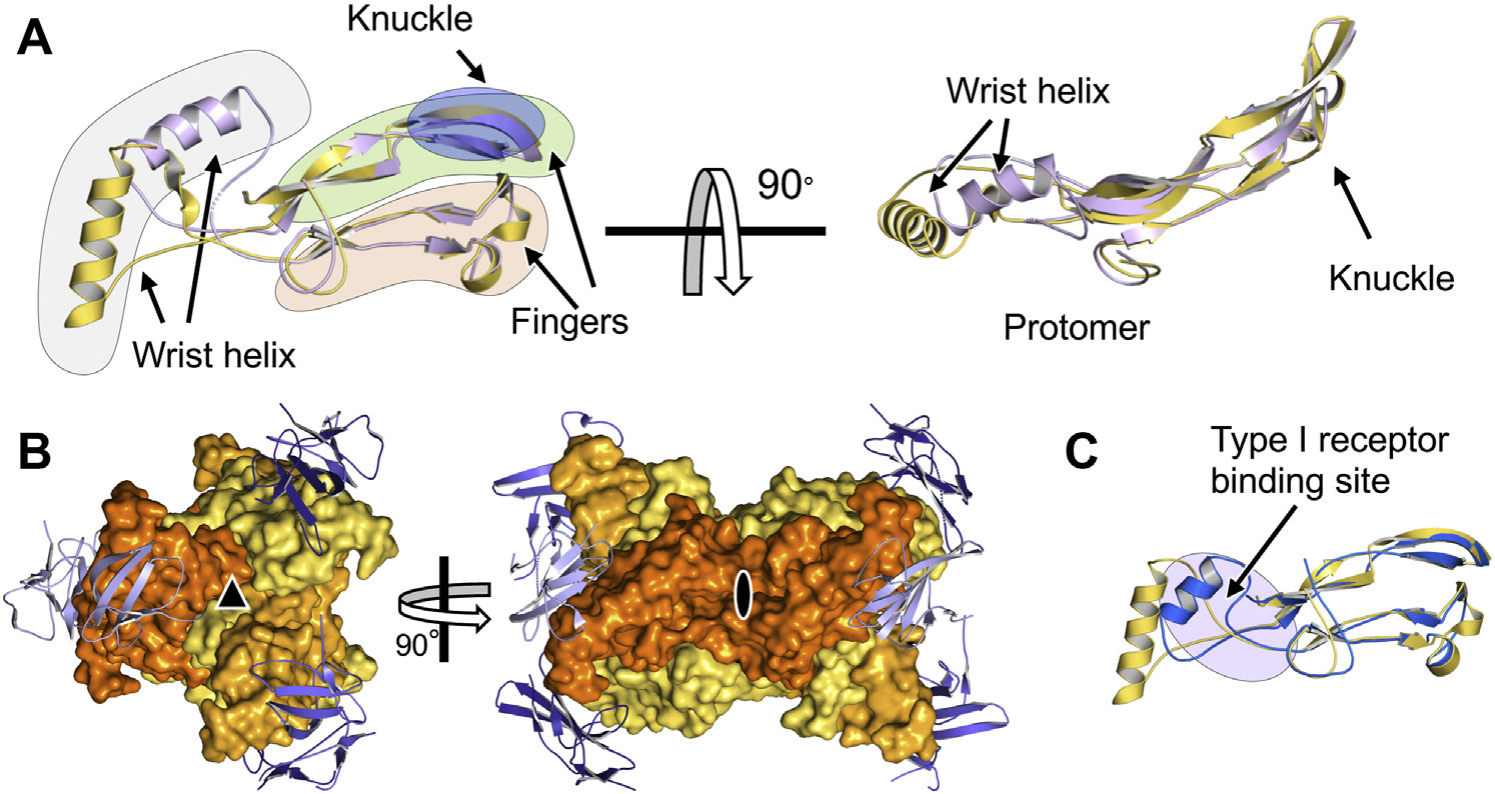
**GF conformations.***A*, superposition of activin B (*gold*) and activin A (*light purple*) protomers. Previously defined features of the GF structure are highlighted, including the fingers, the wrist helix, and the knuckle epitope. Activin B and A have highly superimposable structures. However, the wrist helix adopts GF specific conformations. *Left* and *right panels* represent orthogonal orientations. *B*, activin B forms a higher order, hexameric complex through crystal contacts. The *left panel* shows a noncrystallographic pseudo-threefold axis. The *right panel* shows a crystallographic two-fold axis that generates the biological dimer. *C*, GDF-11 (*blue*) from the GDF-11–ACVR2B–ALK5 signaling complex (6MAC) is superimposed on activin B (*gold*). The Type I receptor-binding site near the wrist helix is highlighted by the *purple circle*. The wrist helix acquires a distinct orientation in the hexameric-signaling complex. GDF, growth and differentiation factor; GF, growth factor.

GFs are homodimeric or heterodimeric proteins. Protomers are often described as having a hand-like shape, as they contain two sets of antiparallel β-strands with finger-like extensions (the “fingers”) that protrude from a central α-helix (the “wrist helix”) (Fig. 2*A*) (40). Activins exhibit significant flexibility in the wrist helix position (Fig. 2*A*). The relative orientation of the two protomers within the dimer varies significantly in these and other activin structures as a result (Fig. 1, *A* and *B*). This flexibility can be explained by the lose tethering of the wrist helix to the rest of the protomer polypeptide chain, as loops connecting the wrist helix to the finger region are mostly disordered in both activin B and activin A structures. Notably, the relative orientation of wrist helix dictates the overall dimer shape as it packs into the concave finger surface of the opposing protomer. The structures presented here, thus, provide additional evidence that the dimer structure of activin class ligands is flexible and that the wrist helix can exist in multiple conformations, leading to significant variability in dimer conformation. We speculate that such conformational plasticity could enable activins to bind two type II receptors with high affinity, even as their relative orientation on the membrane fluctuates.

The buried surface area of the GF dimer is substantial (Table 1), as has been observed (13, 14), and mainly involves interactions between the wrist helix of one protomer and the inward facing finger surface of the opposite protomer. We also observed a second, substantial interface, which results in the formation of a GF hexamer (Fig. 2*B*). While this contact forms part of a crystallographic interaction, its size could hint at the existence of a higher order activin B oligomer. Notably, type I receptors interact with the wrist helix at the GF dimer interface (Fig. 2*C*) (18, 20, 21, 39) and this feature is occluded in the hexameric activin B complex. Thus, activin B may not be able to interact with type I receptors in the form observed here. In addition, the orientation of the wrist helix in this complex is unique compared with homologous GFs that are bound to type I receptors (18, 20, 21), indicating activin B must acquire a different conformation from that observed here to bind type I receptors in the same way as other GFs.

### Conserved hydrophobic hot spot and variable loops form GF interaction site

**Figure 3:**
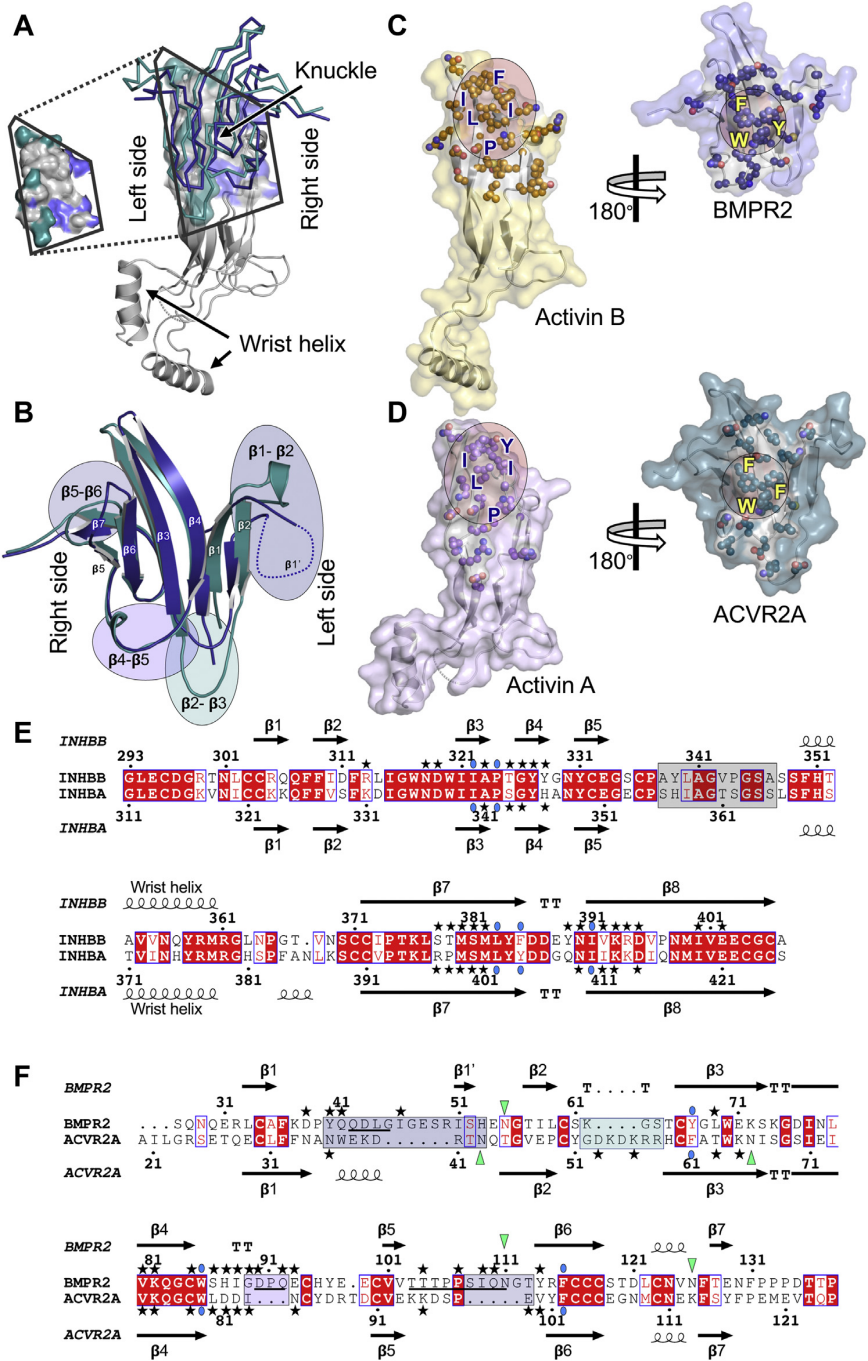
**The receptor–GF interaction.***A*, superposition of the GF fingers shows the orientation of BMPR2 (*blue*) and ACVR2A (*teal*) on their respective GF is analogous. The interaction surface reveals some areas are contacted by both receptors (*gray*), whereas others are closer to BMPR2 (*blue*) or ACVR2A (*teal*). The *inset* highlights contact area preferences. *B*, least squares superposition of BMPR2 (*blue*) and ACVR2A (*teal*). Main chain atoms of β-strands 3 and 4 were used in the alignment. β-strands and loops connecting anti-parallel β-strands (*i.e.*, finger loops) are labeled. Finger loops correspond to most variable sequences in BMPR2 and ACVR2A. *Circles* show finger loops, with *blue circles* highlighting the longer BMPR2 loops and the *green circle* highlighting the longer ACVR2A loop. With exception of the β1–β2 loop, longer loops generally lead to more GF contacts. *C*, BMPR2–activin B complex. Activin B and BMPR2 are shown as *yellow* and *blue surfaces*, respectively. *D*, ACVR2A–activin A complex. Activin A and ACVR2A are shown as *purple* and *teal surfaces*, respectively. The views in (*C*) and (*D*) correspond to the orientation in (*A*), with the GF on the *left side* and the receptor rotated by 180 degrees on the *right side*. Contact areas are highlighted by the *red circles*. All residues within contact distance are shown as atoms. The main aliphatic GF and hydrophobic receptor contact residues are also labeled. *E*, sequence alignment of activin B and activin A. These GFs share 64.3% sequence identity over 115/116 amino acids. *F*, sequence alignment of BMPR2 and ACVR2A. In both alignments, *red areas* indicate sequence identity. *Stars* indicate residues that are within contact distance of their interacting partner. *Blue circles* denote conserved contact residues labeled in (*C*) and (*D*). Contact residues were identified using the PISA (60). *Squares* (colored as in *B*) highlight loop areas with significant variability in sequence and disorder in structures. *Green triangles* show receptor GBD N-glycosylation sites. BMP, bone morphogenetic protein; GBD, GF-binding domain; GF, growth factor.

Superposition of the activin A/activin B finger region shows that BMPR2 and ACVR2A bind the knuckle region with nearly identical contacts and orientations (Fig. 3*A*) (41). This conclusion is supported by the near perfect superposability of the central receptor β-strands after alignment of the GF moiety. Gray areas of the GF-interaction surface further highlight shared surface elements recognized by both receptors. Nevertheless, receptors appear to have distinct laterality preferences for GF binding. The interface area indicates in general terms that the BMPR2-interaction surface is weighted toward the right side of the GF and the ACVR2A-interaction surface is weighted toward the left side of the GF. The inserted view of the binding interface underscores this preference.

Structural superposition of the two receptors further indicates that the laterality preference could result from extended loops that vary in length and placement by receptor (Fig. 3*B*). Thus, BMPR2 has three extended loops linking strands β1–β2, β4–β5, and β5–β6 (blue circles), whereas ACVR2A has one extended loop linking strands β2–β3 (green circle). Although these loops are flexible and partially disordered in the BMPR2 structure, they are mostly within contact distance of the GF and, thus, can provide a significant number of unique contacts. Extended BMPR2 loops are generally in position to contact the right side and top of the GF (blue), whereas the extended ACVR2A loop connecting strands β2 and β3 can contact the left side of the GF (green). However, it is also possible that the laterality bias observed here partly results from limited diffraction.

Analysis of the BMPR2–activin B and ACVR2A–activin A interfaces (Fig. 3, *C* and *D*) reveals that most interacting residues are conserved and hydrophobic, with four aliphatic residues and a proline forming the central contact site on activin B and activin A and three aromatic residues forming the hydrophobic hot spot on BMPR2 and ACVR2A. Based on its conservation, we speculate that the aromatic hot spot underlies GF-binding promiscuity of the two receptors and ACVR2B. In contrast to the central, hydrophobic hot spot, peripheral residues are less conserved and enriched in polar or charged amino acids. Structure-based sequence alignments (42) of the two GFs (Fig. 3*E*) and receptors (Fig. 3*F*) further indicate that contact residues are conserved in position but not in sequence, with loops providing most unique contacts. Notably, loop residues immediately following strand β4 (*i.e.*, BMPR2 86–92 and ACVR2A 80–84) contribute extensively to the GF interaction. As these residues are not conserved between the two receptors, we speculate that they could have a key role in establishing receptor selectivity.

### BMPR2 exhibits conformational plasticity and binds a distinct set of GFs

**Figure 4:**
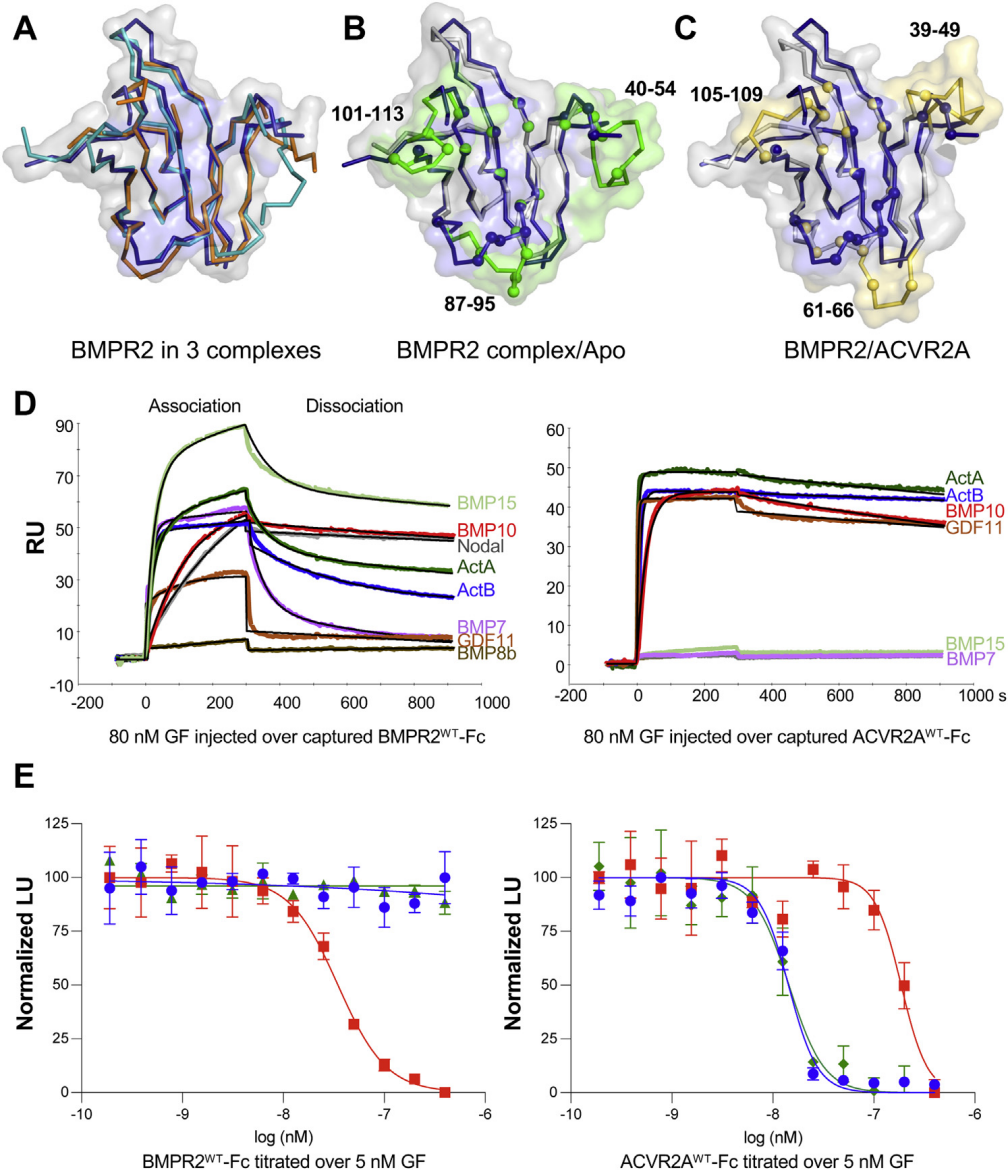
**Structural and functional comparison of BMPR2 and ACVR2A.***A*, superposed noncrystallographic BMPR2 protomers shown as ribbons (*blue*, *cyan*, and *orange*). The surface of one protomer is shown in *gray* with the GF interaction surface colored *blue*. Disordered loops and variability in strand positioning indicate structural flexibility. *B*, superposed APO- (*gray*/*green*, 2HLQ) and GF- (*blue*) bound BMPR2 shown as *ribbons*. APO- and GF-bound BMPR2 surfaces are shown in *gray* with the GF interaction surface colored *blue* and ordered loops in the APO structure colored *green*. Cα atoms of contacting residues are shown as *spheres*, with GF-bound BMPR2 Cα atoms colored *blue* and the corresponding APO BMPR2 Cα atoms colored *green*. Loop regions are ordered in the APO structure (2HLQ) and the finger three loop (β4–β5, residues 87–95) undergoes a significant structural rearrangement upon GF binding. *C*, superposed GF-bound ACVR2A (*gray*/*yellow*) and BMPR2 (*blue*) shown as *ribbons*. BMPR2/ACVR2A surfaces are shown in *gray* with the GF interaction surface of BMPR2 colored *blue* and ACVR2A loops colored *yellow*. Cα atoms of the respective GF contacting residues are shown as *spheres*, with BMPR2 contact residues colored *blue* and ACVR2A contact residues colored *yellow*. The ACVR2A finger two loop (β2–β3, residues 61–66) extends relative to the BMPR2 and provides a distinct set of GF contacts. *D*, GF-binding specificity determined by SPR. BMPR2-Fc (*left panel*) and ACVR2A-Fc (*right panel*) were captured on an SPR sensor chip. 80 nM activin A, activin B, Nodal, BMP7, BMP8b, BMP10, BMP15, or GDF11 were injected over the bound receptors. BMP10 and Nodal bind BMPR2 with high affinity as indicated by the slow dissociation rates. Samples are color coded as noted in the figure, *black lines* correspond to the fitted curved. *E*, GF inhibition by BMRP2-Fc and ACVR2A-Fc. Stably transfected HEK293 reporter cells were induced with 5 nM BMP10, activin B, or activin A. Increasing concentrations of BMRP2-Fc inhibit BMP10 (*red*) (*left panel*). Increasing concentrations of ACVR2A-Fc inhibit BMP10 (*red*), activin B (*blue*), or activin A (*dark green*) (*right panel*). Luciferase units are normalized to GF control (Normalized Luciferase Units, Normalized LU). The average of three biological replicates is shown with error bars representing SD. BMP, bone morphogenetic protein; GDF, growth and differentiation factor; GF, growth factor; HEK293, human embryonic kidney cells.

Crystals obtained here contained multiple GF–receptor complexes in the asymmetric unit, allowing us to evaluate the dynamic nature of the various components. Superposition of the GFs indicates GF protomers have a relatively fixed structure, as they are nearly identical except for the activin A wrist helix orientation (Fig. S3). Similarly, receptor positioning on the GF did not diverge much between BMPR2 and ACVR2A with respect to the placement of contact residues and orientation of the contacting β-strands, indicating a significant degree of functional and structural conservation in the receptor–GF interaction (Fig. S4). In addition, the different ACVR2A structures were completely superimposable, indicating that the conformational flexibility of the ACVR2A GBD is limited (Fig. S5*A*). By contrast, BMPR2 exhibited significant variability in loop regions and in areas that were not directly involved contacting the GF (Figs. 4*A* and S5*B*), possibly reflecting the intermediate-binding affinity of BMPR2 for activin B (28) and the conformational plasticity of the GF-free BMPR2 structures (23). In fact, loop conformations in the GF-free structure would preclude GF binding, supporting the idea that these loops adopt distinct conformations in the GF-free and GF-bound forms (Fig. 4*B*) (23). Notably, these mobile loops are shorter and generally better ordered in the GF-bound ACVR2A, where they contribute significantly to the GF interaction (Fig. 4*C*). Our findings together with previous structural analysis, therefore, indicate that the BMPR2 GBD is inherently flexible and dynamic. This idea is further supported by the higher average B-factor of BMPR2 (∼175 Å^2^) relative to activin B (∼115 Å^2^) in this complex.

Although BMPR2 and ACVR2A utilize a similar GF-binding mode and employ a conserved set of GF-interacting residues, they have distinct GF-binding specificities (8, 28, 43). To better define GF utilization by the two receptors, we evaluated binding of several GFs that have been shown to interact with BMPR2 (28, 29, 35, 36). We were able to obtain good estimates of binding rates and equilibrium-binding constants using single injection surface plasmon resonance (SPR) at room temperature and we carried out titrations over several concentrations to confirm kinetic parameters for GFs that are relevant to this work (Figs. 4*D* and S6, Table 2). With exception of BMP8b and GDF11, all tested GFs bound soluble BMPR2 GBD-Fc. BMP10 and Nodal had the most stable interaction as indicated by their slow dissociation rate, while activin A, activin B, and BMP15 had similar interaction kinetics with fast association and dissociation rates. BMP7 presented a unique example as it dissociated from BMPR2 at a very fast rate. In contrast to BMPR2, ACVR2A GBD-Fc bound fewer of the tested GFs. They included BMP10, activin A, and activin B, as well as GDF11. Notably, the GF-binding spectrum of ACVR2A was more stratified, with activin A, activin B, and GDF11 forming extremely stable complexes (Table 2). These results support previous data showing that ACVR2A and BMPR2 bind an overlapping group of ligands (28); however, ACVR2A binds activin type GFs, including GDF11, with much higher affinity than other tested GFs. We note that a new BMP7 formulation from RnD systems likely accounts for differences with a previous study (28).

**Table 2:** SPR-based GF–receptor-binding rates

Binding parameters	BMP7	BMP8b	BMP10	BMP15	GDF11	Nodal	ActA	ActB
BMPRII-Fc (single)								
*k*_a_	5.2E+05	*NB*	9.1E+04	3.3E+05	*NB*	4.2E+04	3.7E+05	7.9E+05
*k*_d_	7.1E-03	*NB*	1.9E-04	2.1E-03	*NB*	1.4E-04	3.6E-03	1.8E-03
*K*_*D*_	1.3E-08	*NB*	2.1E-09	6.3E-09	*NB*	3.2E-09	9.6E-09	2.3E-09
SD *K*_*D*_	4.4E-09	*NB*	2.8E-10	1.0E-09	*NB*	2.8E-11	1.5E-09	2.5E-10
ACVR2A-Fc (single)								
*k*_a_	*NB*	*NB*	3.6E+05	*NB*	3.5E+06	*NB*	2.1E+06	1.4E+06
*k*_d_	*NB*	*NB*	3.5E-04	*NB*	1.8E-04	*NB*	1.9E-04	7.4E-05
*K*_*D*_	*NB*	*NB*	1.0E-09	*NB*	5.2E-11	*NB*	9.0E-11	5.3E-11
SD *K*_*D*_	*NB*	*NB*	3.0E-10	*NB*	8.3E-12	*NB*	4.3E-12	1.0E-11


	BMPRII-Fc (titration)		ACVR2A-Fc(titration)	
	ActB	BMP10	ActA	ActB
*k*_a_	8.8E+05	2.3E+04	6.0E+05	6.1E+06
*k*_d_	6.2E-04	5.5E-05	1.2E-05	9.7E-06
*K*_*D*_	7.1E-10	2.4E-09	1.8E-11	1.6E-12
Chi^2^	0.93	0.15	1.72	1.93

*k*_a_ (M^−1^ s^−1^), *k*_d_ (s^−1^), *K*_*D*_ (M), *NB*, no binding, single: single curve fit averages of two independent injections 80 nM GF injections, titration: global fit of 2.5 nM, 5.0 nM, 10.0 nM, 20.0 nM, and 40.0 nM GF injections.

To uncover functional consequences of the receptor–GF interaction, we tested the ability of the GBD-Fc fusions to inhibit signaling by activin A, activin B, and BMP10 (Fig. 4*E* and Table 3). Using stably transfected human embryonic kidney (HEK293) reporter cells, we found increasing concentrations of ACVR2A GBD-Fc–inhibited both activin A/B and BMP10 signaling, as indicated, respectively, by the reduced SMAD2/3 and SMAD1/5/8 responses. By contrast, BMPR2 GBD-Fc inhibited BMP10 but not activin A or activin B signaling. This result may appear surprising, as both receptor fusions bind the four GFs with appreciable affinity. However, in the context of cells that express ACVR2A and are grown at 37 °C, BMPR2-Fc may not bind activins with high enough affinity to compete with the much higher affinity ACVR2A interaction. Along the same lines, BMPR2 GBD-Fc inhibited 5 nM BMP10 with greater potency than ACVR2A GBD-Fc (34.7 and 187.0 nM, respectively, Table 3), although both receptor fusions bind BMP10 with similar affinities (Table 2) (20, 28). These results indicate how GF signaling could be affected by cell-specific receptor levels (44, 45).

**Table 3:** Inhibitory potency of Fc Fusions

Receptor	BMP10	Activin B
BMPRII-Fc	3.5E-08	*NI*
ACVR2A-Fc	18.7E-08	1.4E-08

IC_50_ (M), *NI*, no inhibition.

### Hydrophobic hot spot variants identified in PAH patients lack GF-binding activity

**Figure 5:**
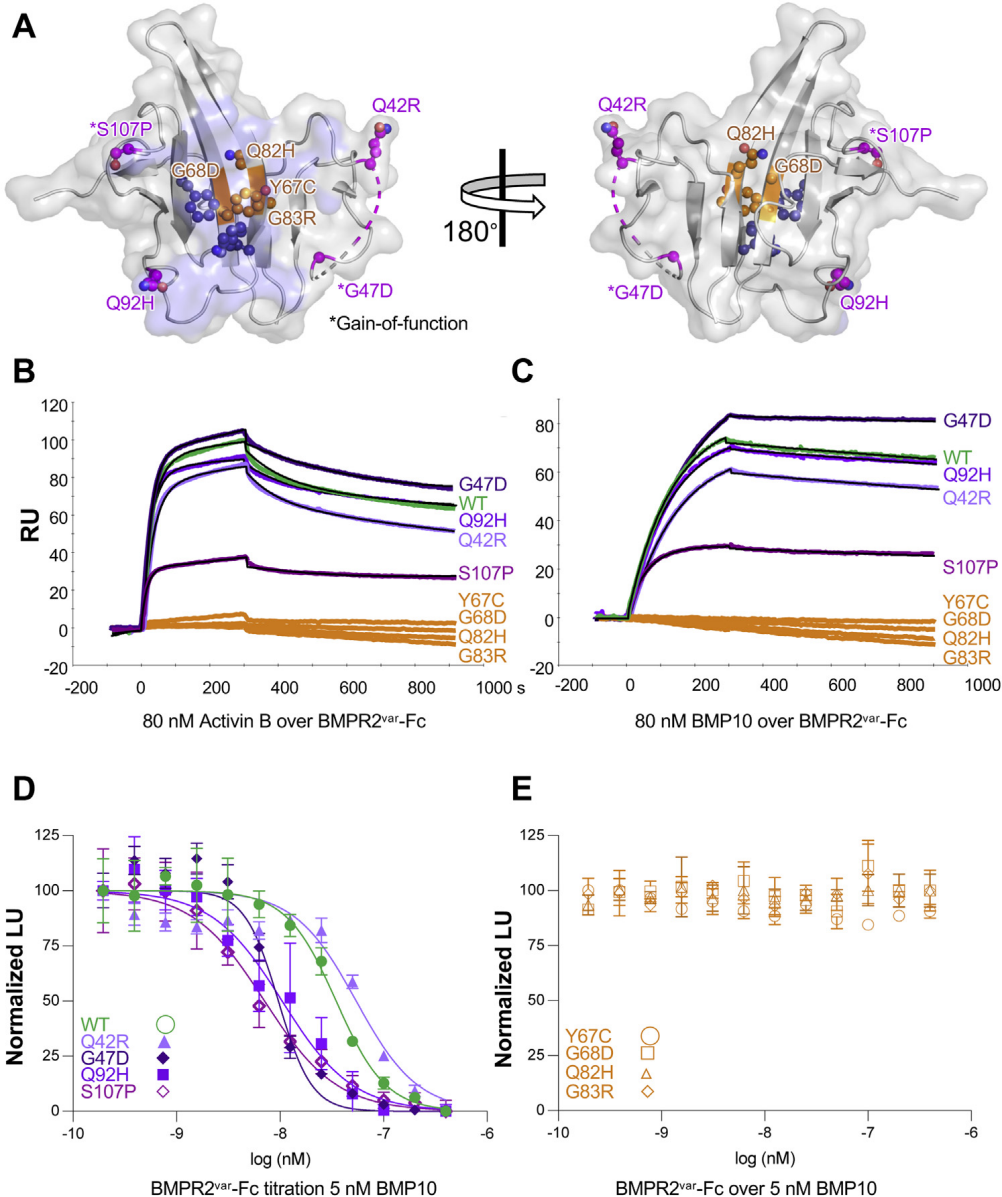
**BMPR2 growth factor–binding domain variants in PAH.***A*, GF-bound BMPR2 GBD shown with residues mutated in PAH. *Orange* colored residues are near or form part of the hydrophobic hot spot. *Magenta* colored residues are on the periphery of the GF-binding interface. *Asterisks* mark gain-of-function variants. *Dark purple*-colored residues form part of the hydrophobic hot spot. The *blue surface* corresponds to the GF-binding interface. *Left* and *right panels* are related by a 180-degree rotation. *B* and *C*, GF-binding properties of PAH variants analyzed by SPR. BMPR2-Fc variants were captured by an SPR sensor chip. 80 nM activin B (*B*) or BMP10 (*C*) were injected over the captured variants. WT BMPR2 is shown in *green*, sensograms corresponding to binding site variants are shown in *orange*, and sensograms corresponding to peripheral variants are shown in *purple*. All binding site variants lose their GF-binding function. By contrast, peripheral variants bind the two tested GFs similar to WT BMPR2. Samples are color coded as noted in the figure, *black lines* correspond to the fitted curved. *D* and *E*, GF inhibition by BMRP2-Fc variants. Stably transfected HEK293 reporter cells were induced with 5 nM BMP10. Increasing concentrations of BMRP2-Fc peripheral (*D*) and hot spot (*E*) were added. Peripheral variants Q42R, G47D, Q92H, and S107P inhibit BMP10 signaling, whereas binding site variants Y67C, G68D, Q82H, and G83R do not. Luciferase units are normalized to GF control (Normalized Luciferase Units, Normalized LU). The average of three biological replicates is shown with error bars representing SD. BMP, bone morphogenetic protein; GBD, GF-binding domain; GF, growth factor; HEK293, human embryonic kidney cells; PAH, pulmonary arterial hypertension.

Several BMPR2 GBD missense mutations have been identified in PAH patients (Table 4) (31). Variants could be categorized as forming part of the GF interaction surface (orange), of peripheral loop areas (purple), or of the conserved disulfide core (Fig. 5*A*). We produced multiple variants as Fc fusions using stably transfected CHO cells as described. We excluded disulfide core variants as they are expected to misfold. Purified variants were monodisperse as determined by size-exclusion chromatography (SEC) and eluted at a volume consistent with the expected molecular weight (Fig. S7), indicating they were properly folded. Using SPR, we tested binding of variants to activin B and BMP10 (Figs. 5, *B* and *C* and S8). Variants that formed part of the GF interaction surface failed to bind BMP10 and activin B, as indicated by the absent SPR response (orange curves). These results provide direct evidence that the hydrophobic hot spot is critical for GF binding and that the crystal structure is biochemically relevant. By contrast, peripheral loop variants (purple curves) bound activin B and BMP10 with affinities that were comparable to those of the WT receptor (green curve), indicating that the contribution of these loops to GF-binding affinity and specificity may be relatively modest and possibly indirect. Notably, variants that potentially have increased loop rigidity (G47D and S107P) exhibited a modest gain in binding affinity relative to the WT receptor, as reflected in the lower equilibrium dissociation constant (*K*_*D*_, Table 5).

**Table 4:** PAH mutations in BMPR2 GBD

Orange: hydrophobic hot spot residues or residues near the hydrophobic hot spot. Purple: peripheral loop residues. Black: cysteines involved in disulfide bond formation are colored black. Abbreviation: DS, Disulfide Cysteine.

**Table 5:** Variant binding rates and inhibitory potency

Binding parameters	WT	Q42R	G47D	Q92H	S107P
BMP10 (single)					
*k*_a_	9.4E+04	8.3E+04	6.5E+04	9.0E+04	2.1E+05
*k*_d_	1.7E-04	2.1E-04	3.8E-05	1.5E-04	1.8E-04
*K*_*D*_	1.8E-09	2.5E-09	5.8E-10	1.8E-09	8.6E-10
*SD K*_*D*_	1.4E-10	3.0E-10	1.5E-10	1.3E-10	6.4E-12
BMP10 (titration)					
*k*_a_	2.3E+04	3.4E+04	1.4E+05	2.5E+05	1.9E+04
*k*_d_	5.5E-05	7.9E-05	2.8E-06	7.8E-05	1.9E-05
*K*_*D*_	2.4E-09	2.3E-09	2.0E-11	3.2E-10	1.0E-09
Chi^2^	0.15	0.03	0.027	0.22	0.08
IC_50_	3.5E-08	5.4E-08	1.0E-08	1.0E-08	0.7E-08
Activin B (single)					
*k*_a_	4.7E+05	4.2E+05	4.4E+05	4.0E+05	6.1E+05
*k*_d_	1.8E-03	2.1E-03	1.3E-03	7.2E-04	1.5E-03
*K*_*D*_	4.1E-09	5.0E-09	2.9E-09	1.8E-09	2.6E-09
*SD K*_*D*_	1.1E-09	8.9E-10	3.5E-10	7.8E-11	5.8E-10
Activin B (titration)					
*k*_a_	8.8E+05	5.8E+05	4.3E+05	4.0E+05	6.7E+05
*k*_d_	8.2E-04	8.2E-04	5.8E-04	4.4E-04	1.9E-04
*K*_*D*_	7.1E-10	1.4E-09	1.3E-09	1.1E-09	2.8E-10
Chi^2^	0.93	1.46	1.76	1.93	0.30
IC_50_	*NI*	*NI*	*NI*	*NI*	*NI*

*k*_a_ (M^−1^ s^−1^), *k*_d_ (s^−1^), *K*_*D*_ (M), IC_50_ (M), *NI*, no inhibition, single: single curve fit averages of two independent injections 80 nM GF injections, titration: global fit of 2.5 nM, 5.0 nM, 10.0 nM, 20.0 nM, and 40.0 nM GF injections.

To validate our SPR results, we tested BMP10 signaling inhibition by the different GBD-Fc variants using stably transfected HEK293 reporter cells. Consistent with the SPR data in Figure 5*C*, we found that peripheral loop variants inhibited BMP10 signaling (Fig. 5*D*), whereas interaction surface variants did not (Fig. 5*E*). Three variants (G47D, Q92H, and S107P) inhibited BMP10 signaling with significantly greater potency than WT BMPR2 GBD-Fc (Table 5). Notably, PAH is generally assumed to be caused by BMPR2 loss of function. We show here that most GBD variants fit that model. However, we identify four variants that retain or gain GF-binding activity, indicating that these variants either promote PAH pathogenesis by a distinct mechanism or that they are mischaracterized as pathogenic (Table 4).

## Discussion

We have solved the crystal structures of the type II BMPR2 GBD in complex with activin B and of the type II activin Receptor ACVR2A in complex with activin A. We show that BMPR2 and activin type II receptors interact with GFs using a nearly identical mode of binding. Placement of the two receptors on their respective GFs is analogous in geometry, as central secondary structural elements of both type II receptors as placed on the GFs superpose well. Receptor–GF interactions are mostly preserved as both receptors contact a conserved set of aliphatic GF residues using a conserved set of aromatic residues that form a central hydrophobic hot spot. However, differences between receptors are also apparent. Most notably, the length and sequences of the loops that extend from the various “fingers” allow for unique receptor–GF contacts. These are weighted toward different sides of the GF, with BMPR2 favoring contacts with the right side of the GF and ACVR2A favoring the left side. In addition, BMPR2 residues 86 to 92 and ACVR2A residues 80 to 84, which form part of the loop connecting strands β4 and β5 and immediately follow the hydrophobic hot spot W85 (BMPR2) or W79 (ACVR2A), contribute significantly to the GF interaction but differ in sequence, highlighting a region that could account for differences in GF recognition between the two receptors. Collectively, these results indicate how receptors could acquire distinct specificities within the framework of a highly conserved, central interaction hot spot. Variations in peripheral loop residues could provide a platform of unique contacts to establish receptor–GF binding selectivity.

ACVR2A and BMPR2 can potentially interact with the same 30 GFs (8, 9, 10, 11). Although their distinct biological functions indicate differences in their GF binding and signaling activities (46), (coma) questions about their GF-binding selectivity remain. Here, we demonstrated that the BMP15, BMP10, and Nodal are the highest affinity BMPR2 GFs. By contrast, activin A, activin B, and GDF11 are the highest affinity ACVR2A GF. Nevertheless, BMP10 also bound ACVR2A, and activins A and B also bound BMPR2 with considerable affinity, revealing a significant overlap in receptor utilization by these GFs. However, the faster dissociation rate of activins A and B from BMPR2 indicated that activin–BMPR2 complexes are less stable than activin ACVR2A complexes, providing a rationale for the preferential utilization of ACVR2A by activins. These observations were mirrored when analyzing signaling by the three GFs and their inhibition by the traps BMPR2-Fc and ACVR2A-Fc. Both traps inhibited BMP10, potentially reflecting the comparable binding affinity of BMPR2 and ACVR2A for BMP10. By contrast, only ACVR2A-Fc inhibited activin A and activin B, suggesting that the exceptionally high affinity of activins for ACVR2A could preclude their inhibition by the lower affinity interactor BMPR2 GBD-Fc at tested concentrations and temperatures. These observations indicate that in a physiological context, activins preferentially associate and signal *via* their high affinity receptors ACVR2A and ACVR2B. By contrast, BMPs could utilize the three type II receptors ACVR2A, ACVR2B, and BMPR2 equally as they associate with the three receptors with similar affinities. But activins could also signal *via* BMPR2 as shown previously, perhaps in cells that do not express ACVR2A/B (29, 36).

The biological relevance of BMPR2 stems in part from its link to PAH (31). Over 250 mutations throughout the BMPR2 gene have been identified in PAH patients, including in the extracellular GBD region. Most PAH mutations result in loss of BMPR2 function or reduced BMPR2 levels. They include mutations in the GBD cysteines that form the obligate cysteine disulfide core. However, missense mutations in the GBD of unknown consequence have also been identified. We generated these GBD variants and tested their ability to bind and/or inhibit signaling by activin B and BMP10. All mutations within the GF-binding interface resulted in loss of function, indicating that the crystal structure represents a biological complex. Y67C directly contacts the GF, highlighting the importance of the hydrophobic hot spot residues in GF binding. Other GF-binding interface variants are adjacent in sequence or space to Y67C but point away from that site. That these variants fail to bind GF indicates that the overall integrity and shape of the GF-binding interface is critical for GF binding and may be modulated indirectly by amino acids that have a structural rather than functional role. By contrast, all loop variants bound activin B and BMP10, indicating that residues, which are distant in space from the hydrophobic hot spot, may only have a moderate functional significance. Notably, the fact that these variants bind BMPR2 could either indicate that these are misclassified as pathogenic or that they may cause PAH by an alternate mechanism. In fact, three variants gained function as indicated by a slower receptor–GF dissociation rate or by greater inhibitory potency. Two of these variants, G47D and S107P, may lead BMPR2 to have greater structural rigidity, possibly indicating that the inherent flexibility of the BMPR2 GBD may be linked with its overall lower GF-binding affinities. While testing these PAH variants in an *in vitro* signaling assay could clarify if they retain signaling activities, our ability to carry out these experiments is limited as BMP9/BMP10 signal strongly in most standard cell lines, including HepG2, A204, and HEK293 cells (28, 47, 48, 49).

Overall, the structures and biochemistry presented here reveal how the type II TGF-β family receptor BMPR2 binds GFs. These structures demonstrate that ACVR2A, ACVR2B, and BMPR2 use a conserved mode of GF recognition and thus provide a molecular rationale for the promiscuous and shared GF-binding spectrum of the three receptors. But they also reveal interacting regions of low homology that could account for GF selectivity. We further explored biochemical and *in vitro* activities of BMPR2 variants identified in PAH patients. Variants in the binding hot spot are not active, providing direct evidence that the crystal structure represents a biological complex and supporting the idea that BMPR2 loss of function is a key pathogenic mechanism in PAH. Strikingly, PAH variants in peripheral loop areas remained active. This observation either suggests that these variants are misclassified as pathogenic or that they could transform a signaling complex into a loss-of-function complex.

## Experimental procedures

### Receptor Fc fusions

Human BMPR2 cDNA (Q13873) and synthetic genes for human ACVR2A (P27037) and human IgG1-Fc were used to generate the receptor GBD-Fc fusions by two-step PCR. NCBI-protein accession numbers are shown in parentheses. Fusion constructs included the extracellular domains of human ACVR2A (1–120) or BMPR2 (1–138), a 20 amino acid linker containing TEV and enterokinase cleave sites, and a C-terminal Fc. The TEV site immediately followed the receptor GBD and the enterokinase site immediately preceded the Fc moiety. PAH variants were generated from the parental BMPR2-Fc construct *via* two-step PCR. Fusion proteins were expressed in stably transfected CHO cells and purified from conditioned medium (CM) using Protein A capture (MabSelect SuRe, Cytiva) as described (28) followed by SEC in PBS, pH 7.5. SEC removed aggregates ensuring that a monodisperse population of expected apparent molecular weight was used in all downstream studies. Purified proteins were stored at −80 °C.

### Growth factors

Human nodal (Q96S42), BMP7 (P18075), BMP15 (O95972), BMP8b (P34820), and GDF11 (O95390) were obtained from R&D Systems or PeproTech. Activin A (P08476), activin B (Q53T31), and BMP10 (O95393) were produced in-house using stably transfected CHO cells. Activin A was captured from CM by Protein A affinity chromatography. BMP10 was captured from CM by Metal affinity chromatography (Excel, Cytiva). Both GF moieties were separated from the prodomain using Reversed Phase Chromatography (Resource RPC, Cytiva). Activin B was purified as described (50). GFs were lyophilized and stored at −80 °C. NCBI-protein accession numbers are shown in parentheses.

### Crystallization

Receptor GBD were cleaved from the Fc moiety by TEV protease (51, 52), resulting in fragments that consisted of residues 20 to 120 (ACVR2A) and 27 to 138 (BMPR2) followed by the TEV site. Fc was removed by protein A capture. Receptor GBDs were deglycosylated using Endo F3 or PNGase F, purified by SEC, dialyzed into Tris-buffered saline (pH 7.5), and stored at −80 °C. GFs consisted of the full mature moiety, including residues 311 to 426 (activin A) and 293 to 407 (activin B). Both GFs fractions consisted 100% of disulfide linked dimers. For crystallization, GFs were resuspended in 4 mM HCl (RnD Systems, pH ∼ 2.4) and combined with 1.2 M excess receptor GBD. The buffer of the complexes was exchanged by centrifugation to 10 mM Tris–HCl, 40 mM NaCl, pH 8.0. The final concentration of the BMPR2–activin B and ACVR2A–activin A complexes was 11 mg/ml and 9 mg/ml, respectively. Crystallization conditions were identified using the JCSG+ screen (Qiagen). Diffraction quality crystals were obtained after optimizing conditions. BMPR2–activin B crystals were grown in 14% PEG 3K, 175 mM (NH_4_)_2_ SO_4_, 100 mM BisTris, pH 5.4. ACVR2A–activin A were grown in 17% PEG 3K, 100 NaCitrate, pH 5.8.

### Data collection

Crystals for the BMPR2–activin B complex were crosslinked using glutaraldehyde (53), equilibrated in mother liquor containing 30% glycerol, and flash frozen in liquid nitrogen. A 3.45 Å dataset was collected at the Advanced Photon Source, beamline 21-ID-G. Molecular replacement using PHASER (54) placed three activin B protomers in the asymmetric unit. The activin B model was obtained from AlphaFold (38). The BMPR2 GBD (PDBid 2HLR/2HLQ, (23)) with loop regions deleted was placed manually onto one GF protomer, fitted with rigid body refinement, and extended by NCS. Crystals for the ACVR2A–activin A complex were equilibrated in 17% PEG 3K, 100 Na-formate, pH 4.5, and 30% glycerol. Data were processed and reduced with HKL2000 (55). A 3.20 Å dataset was collected on a Rigaku FR-E+ rotating anode generator at 100 K. Data were processed with MOSFLM and reduced with AIMLESS (56, 57). Molecular replacement with PHASER placed four receptor–GF complexes of the ACVR2B–activin A crystal structure in the asymmetric unit (PDBid 1S4Y, (37)). Both structures were refined with PHENIX (58) using NCS restraints for equivalent residues. Manual building was performed in COOT (59). AlphaFold models and high resolution protomer structures were used to assist in model building. Electron density was continuous for all ACVR2A protomers. Activin A and activin B were generally well ordered except for loops connecting the wrist helix. BMPR2 was well defined in regions near the GF; however, loop regions were generally disordered. Water molecules were not placed in the final model. Contact maps and buried surface area values were calculated using the Protein Interfaces, Surfaces, and Assemblies server (60). Structural figures were prepared using PyMOL (61). Atomic coordinates and structure factor amplitudes were deposited in the Protein Data Bank (PDBid 7U5O, 7U5P). For data processing and refinement statistics, see Table S1.

### Surface plasmon resonance

SPR experiments were performed using a BIAcore 3000. Experiments were carried out at 25 °C in HBS/EPS (0.01 M Hepes, 0.5 M NaCl, 3 mM EDTA, 0.005% (v/v) Tween 20, pH 7.4) containing 0.1% bovine serum albumin as running buffer. The experimental flow rate was 50 μl/min. Approximately, 5000 to 7000 Response Units (RU) of Anti-human IgG (Fc) (Human antibody capture kit, Cytiva) were immobilized on three channels of a CM5 chip using amine-coupling chemistry. Approximately, 500 RU of purified BMPR2-Fc WT or BMPR2-Fc variants and 150 RU of purified ACVR2A-Fc were loaded on the experimental flow channels. A reference channel was monitored to account for nonspecific binding, drift, and bulk shifts. To identify ACVR2A- and BMPR2-interacting ligands, 80 nM activin A, activin B, BMP7, BMP8b, BMP10, BMP15, GDF11, and Nodal were injected over the WT receptors. To establish the binding activity of PAH variants, 80 nM BMP10 or activin B was injected over captured BMPR2-Fc^WT^ or BMPR2-Fc^VAR^. The single concentration approach allowed us to obtain an estimate of binding parameters. Injections were repeated two times. Titrations over several concentrations were used to confirm kinetic parameters for GFs and receptors that are relevant to this work. The antibody surface was regenerated to baseline after each binding cycle by injecting MgCl_2_. Sensograms were analyzed by double referencing. To obtain kinetic rate constants, the processed data were fitted to “1:1 binding model” using BiaEvaluation software. The equilibrium-binding constant *K*_*D*_ was determined by calculating the ratio of binding rate constants *k*_*d*_/*k*_*a*_. Results are summarized in Table 1.

### Cell lines

HEK293 were obtained from American Type Culture Collection. Cells were grown in Eagle’s minimum essential medium (EMEM) supplemented with 10% fetal bovine serum and 1% penicillin/streptavidin at 37 °C under 5% humidified CO_2_ conditions as indicated. Passage 5 cells were transfected with the SMAD2/3 responsive reporter plasmid pGL4.48 (luc2P/SMAD binding element (SBE)/HYGRO) or the SMAD1/5/8 responsive reporter plasmid pGL4 (luc2P/2X BMP responsive element (BRE)/PURO) using lipofectamine 2000 and subjected to hygromycin B (50 μg/ml) or puromycin (0.5 μg/ml) selection. Passage 8 SBE reporter cell pools were cryopreserved. BRE reporter cells were subjected to clonal selection and single clones were cryopreserved at passage 11. Freshly thawed cells were passaged twice before seeding 96-well reporter assay plates.

### Reporter assays

Fifty thousand SBE or 10,000 BRE reporter cells per well were seeded in 96-well plates and grown overnight in complete EMEM medium. After 24 h incubation, medium was replaced by assay medium (serum-free EMEM, 5 nM GFs and 0–400 nM receptor-Fc fusion). Assay medium was incubated at room temperature for 1 h before addition to cells. Luciferase expression was measured using a luciferase assay reagent after cells were incubated 16 h in assay medium. Firefly luciferase activity was measured using a FLUOstar Omega plate reader. Reporter gene assays were performed in triplicates and were repeated multiple times. Data presented is the mean of three independent measurements. Error bars represent the SD from three independent measurements. GraphPad Prism 9.3 was used for data fitting, analysis, and for generating graphs.

## Data availability

Data and structures have been deposited at the Protein Data Bank (https://www.rcsb.org) and will be publicly released with publication. Accession numbers of the deposited structures are 7U5O and 7U5P.

## Supporting information

This article contains supporting information.

## Conflicts of interest

E. M.-H. owns, shares, and is a founder/CSO of Advertent Biotherapeutics, Inc. All other authors declare that they have no conflicts of interest with the contents of this article.
