# Supplementary material for: Type II BMP and activin receptors BMPR2 and ACVR2A share a conserved mode of growth factor recognition
Source: J Biol Chem. 2022 May 26;298(7):102076. doi: 10.1016/j.jbc.2022.102076 (PMC9234707; doi:10.1016/j.jbc.2022.102076)
Supplement: Supplemental Table S1 [file mmc1.docx]

**Table S1.** **Data collection and refinement statistics**

|  | BMPR2 – Activin B | | ACVR2A-Activin A |
| --- | --- | --- | --- |
| **Data collection** |  |  | |
| Source | LS-CAT 21-ID-G | Rigaku FR-E+ | |
| Space group | *C*2_1_ | *P*2_1_2_1_2_1_ | |
| Cell dimensions |  |  | |
| *a*, *b*, *c* (Å) | 81.0, 115.65, 110.2 | 82.6, 82.5, 151.3 | |
| α, β, γ (°) | 90.0, 100.9, 90.0 | 90.0, 90.0, 90.0 | |
| Resolution (Å) | 60.34-3.45 (3.78-3.45) | 25.00-3.15 (3.20-3.15) | |
| *R*_merge_ | 0.092 (0.355) | 0.065 (0.473) | |
| *I* / σ*I* | 5.5 (2.8) | 22.8 (2.9) | |
| Completeness (%) | 99.8 (100.0) | 95.4 (90.7) | |
| Redundancy | 5.5 (2.8) | 22.8 (2.9) | |
|  |  |  | |
| **Refinement** |  |  | |
| Resolution (Å) | 50.0-3.45 | 24.68-3.15 | |
| No. reflections | 13115 (1288) | 17763 (1631) | |
| *R*_work_ / *R*_free_ | 23.6/28.4 | 21.7/27.6 | |
| No. atoms |  |  | |
| Protein | 4388 | 6326 | |
| Glycan | - | 116 | |
| Water/ion | - | - | |
| *B*-factors |  |  | |
| Protein | 141.78 | 81.34 | |
| r.m.s. deviations |  |  | |
| Bond lengths (Å) | 0.003 | 0.004 | |
| Bond angles (°) | 0.692 | 1.050 | |
| Ramachandran |  |  | |
| Favored | 93.13 | 93.61 | |
| Outliers | 0.72 | 0.51 | |
| Accession Number | 7U5O | 7U5P | |

*Values in parentheses are for highest-resolution shell.
