# Supplementary material for: Type II BMP and activin receptors BMPR2 and ACVR2A share a conserved mode of growth factor recognition
Source: J Biol Chem. 2022 May 26;298(7):102076. doi: 10.1016/j.jbc.2022.102076 (PMC9234707; doi:10.1016/j.jbc.2022.102076)
Supplement: Supplemental Figures S1–S8 [file mmc2.pdf]

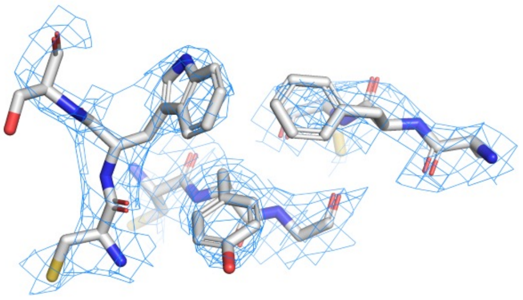

BMPR2 – Activin B

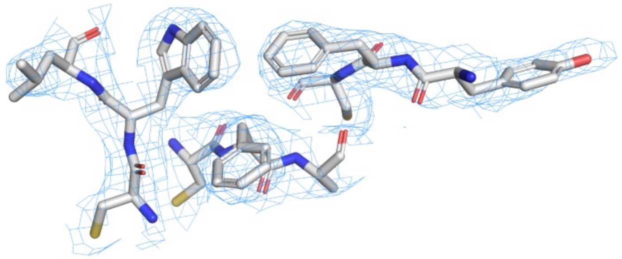

ACVR2A – Activin A

Figure S1: 2Fo-Fc map of hydrophobic hot spot residues. The density of BMPR2 is consistent with a low-resolution structure and a moiety that exhibits a high average B-factor.

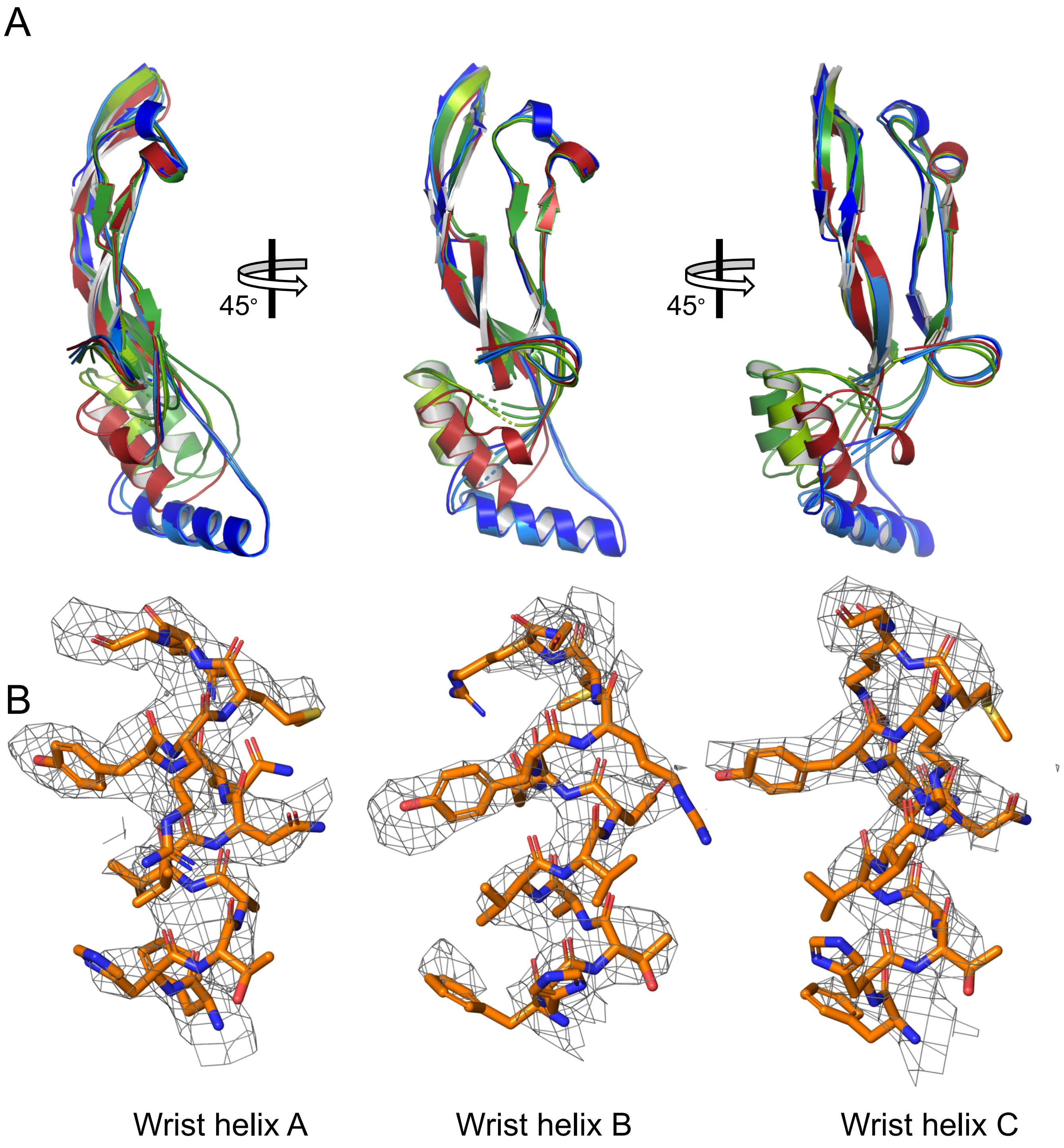

Figure S2: A) Superposition of GF Protomers. The three activin B protomers in the structure are colored blue, three activin A protomers in the structure are colored green, the AlphaFold model used for MR is colored red. Note the significant shift in the wrist helix between the AlphaFold model and the activin B structure after refinement. B) Simulated annealing F_o-F_c map superimposed over the refined activin B wrist helices, corresponding to the three activin B protomers.

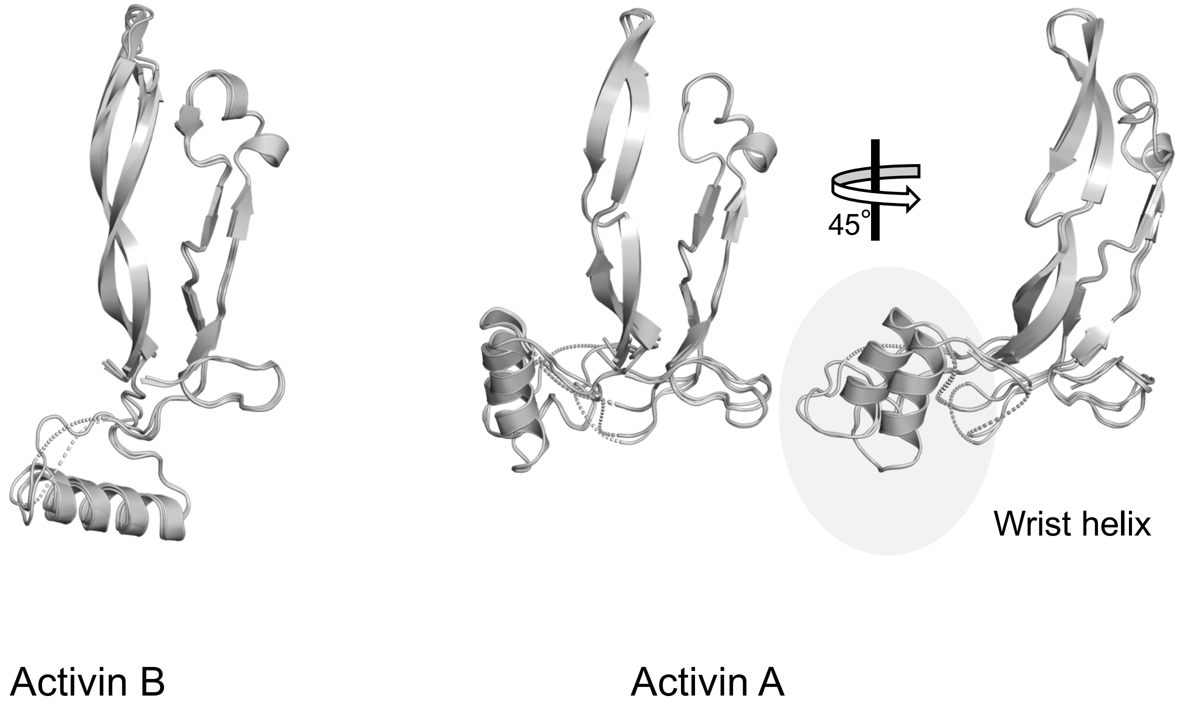

Figure S3: Superposition of GF protomers within asymmetric unit shows the finger region is highly superimposable, as is the Activin B wrist helix. By contrast, the Activin A wrist helix is present in two distinct conformations.

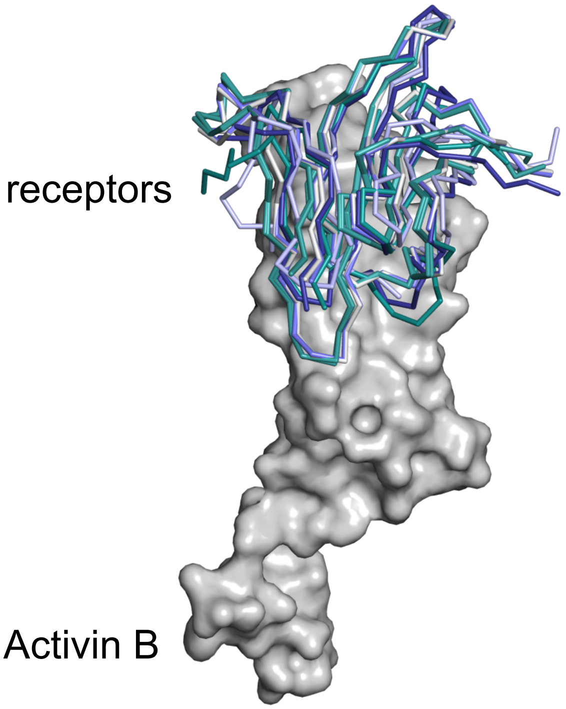

Figure S4: Superposition of all GF protomers. One Activin B and all non-crystallographic receptor protomers. Receptors are placed in a nearly identical position on the GF. The small differences observed between BMPR2 protomers could reflect the low resolution of the structure, structural flexibility of BMPR2, and/or the low affinity of the complex.

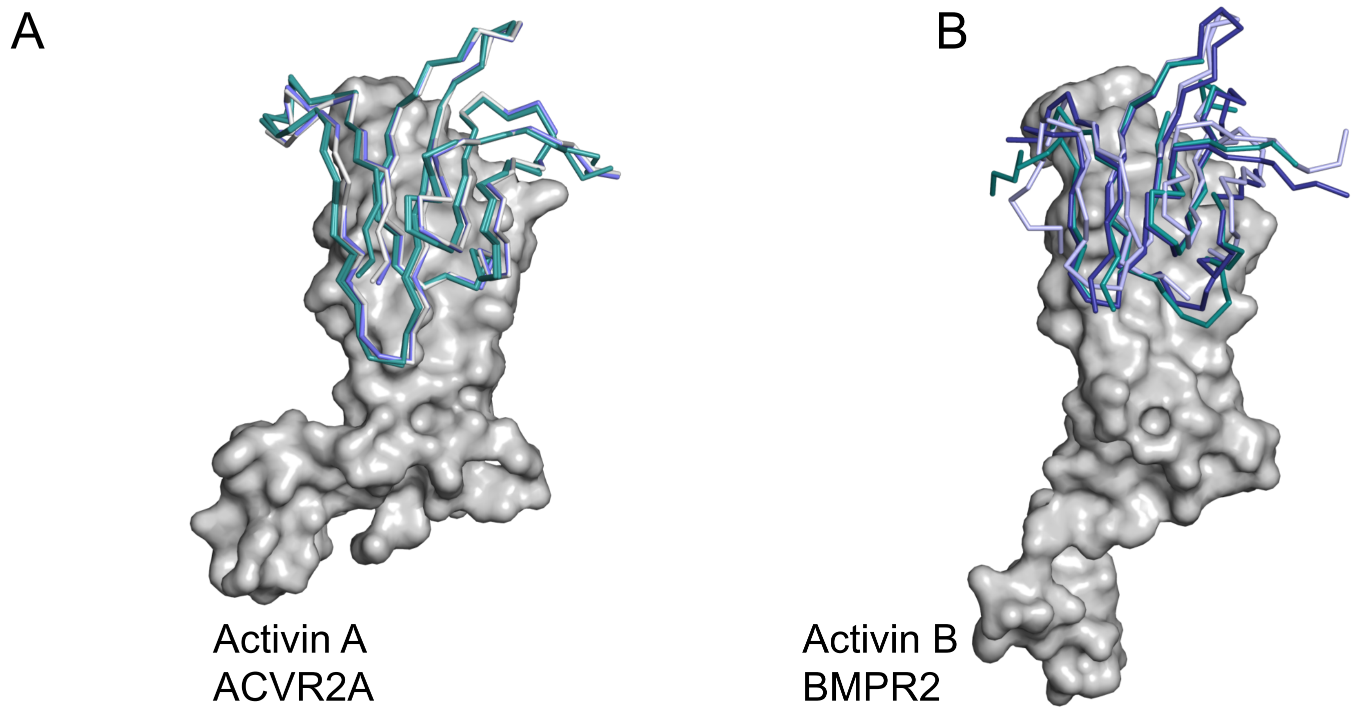

Figure S5: A) Superposition of Activin A protomers. B) Superposition of Activin B protomers. One GF and all non-crystallographic receptor protomers are shown as indicated. Structural plasticity of BMPR2 outside of receptor binding regions can be noted.

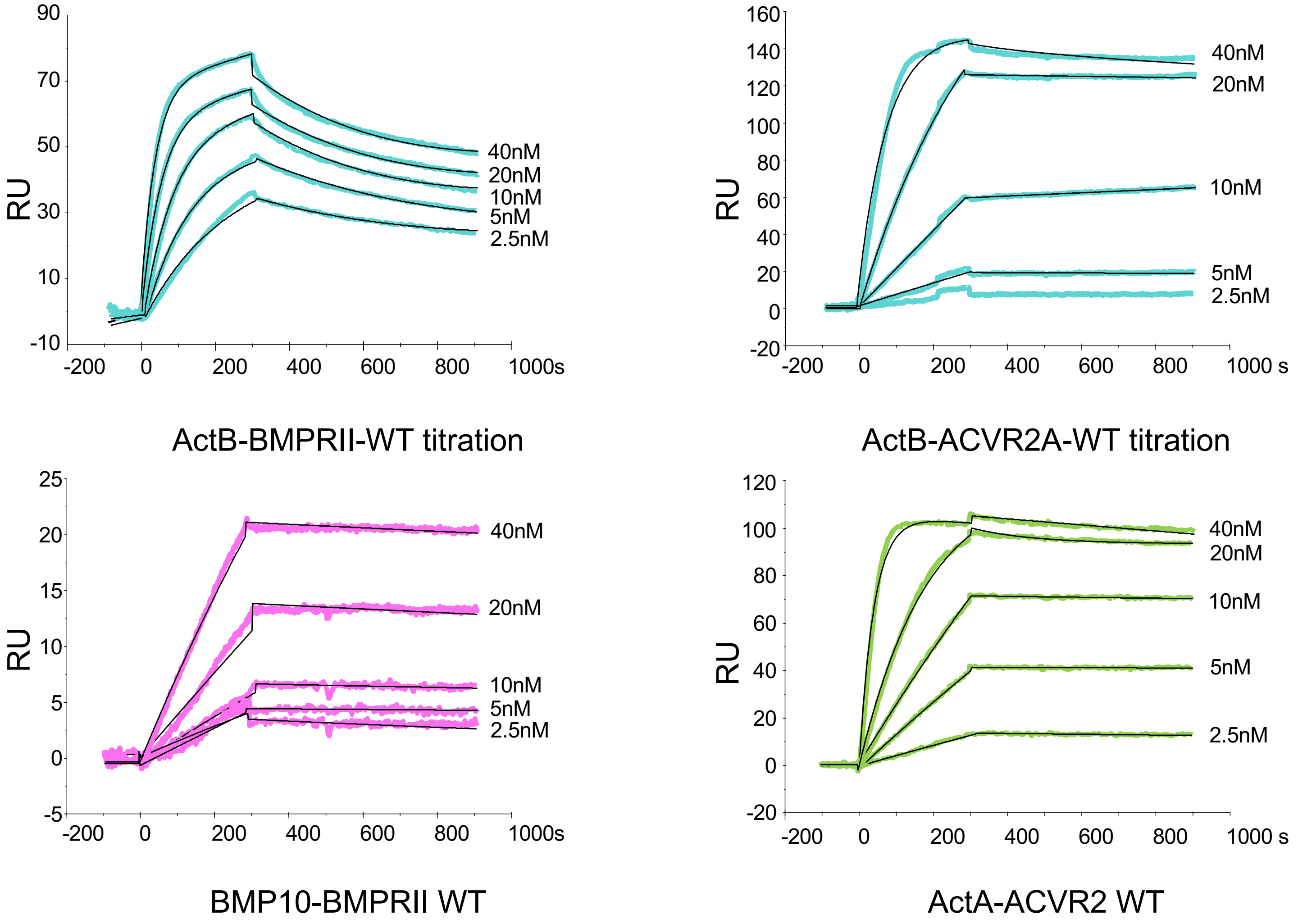

Figure S6: SPR titration of various GFs vs wild-type receptors as noted. Concentrations are shown on the side of each panel. Kinetic parameters are shown in the tables.

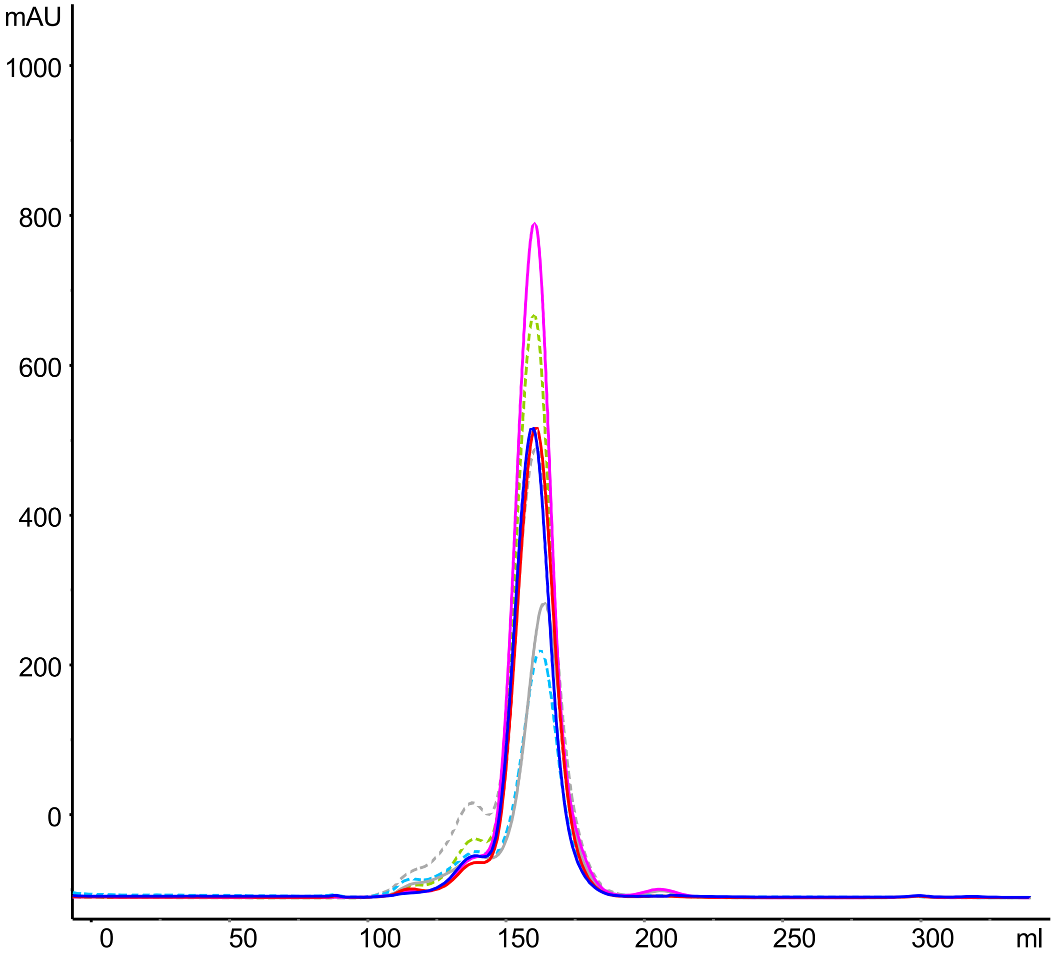

Figure S7: SEC chromatograms of BMPR2^{VAR}-Fc. All BMR2-Fc fusion variants can be purified to homogeneity by size exclusion chromatography, indicating all constructs are properly folded and correspond to the dimeric form.

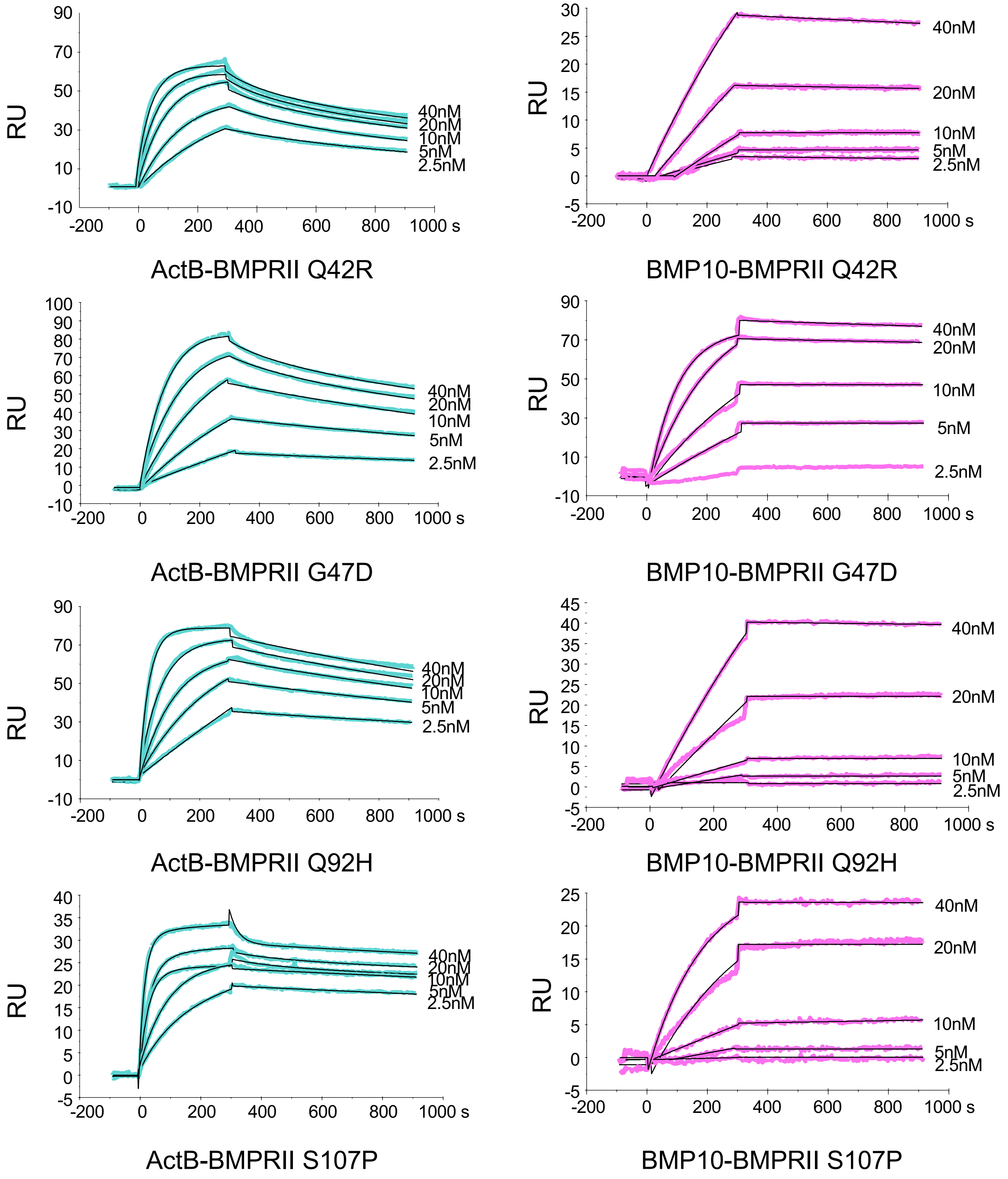

Figure S8: SPR titration of various Activin B (green) and BMP10 (pink vs PAH variants. Concentrations are shown on the side of each panel. Kinetic parameters are shown in the tables.
